# Exposure to Polystyrene Microplastics Disrupts Blood Cell Homeostasis and Metabolic Profiles in Pregnant Mice and Offspring: The Role of Oxidative Stress and Inflammation

**DOI:** 10.3390/toxics14050354

**Published:** 2026-04-23

**Authors:** Lin Lin, Ti-Zhen Yan, Hai-Wen Zhuo, Rong-Hua Zhang, Hong-Yi Liu, Xing-He Wang, Qing-Wo Lu, Rui Guo, Jian-Feng Qiu, Bo Zhang, Qing-Ming Luo

**Affiliations:** 1 School of Public Health, Southern Medical University, Guangzhou 510515, China; linlin_smu@163.com (L.L.); 18819006458@163.com (H.-Y.L.); 2 Dongguan Maternal and Child Health Care Hospital, Dongguan 523000, China; yantizhen@gdmu.edu.cn (T.-Z.Y.); zhuohaiwen@163.com (H.-W.Z.); 13728299529@163.com (R.-H.Z.); wxh130009@163.com (X.-H.W.); lqw850@163.com (Q.-W.L.); 3 National & Local United Engineering Laboratory of Natural Biotoxin, Fuzhou 350002, China; ruiguo@fafu.edu.cn (R.G.); jfqiu@fafu.edu.cn (J.-F.Q.); 4 College of Bee Science and Biomedicine, Fujian Agriculture and Forestry University, Fuzhou 350002, China

**Keywords:** microplastics, pregnancy, oxidative stress, inflammation, metabolomics, candidate biomarkers

## Abstract

Micro/nanoplastics (MNPs) are emerging contaminants of concern for maternal and fetal health, yet their effects on the maternal–fetal circulation and serum metabolic homeostasis remain unclear. Here, we investigated the maternal and offspring toxicity of polystyrene microplastics (PS-MPs) and serum metabolomic alterations in dams and offspring. PS-MPs accumulated in multiple tissues, including blood, indicating maternal-to-offspring transfer. Continuous exposure reduced litter size, induced hepatic oxidative stress, and increased IL-6 and TNF-α levels in a dose-dependent manner in both dams and offspring. In dams, PS-MPs also decreased red blood cell and platelet counts and altered leukocyte composition, with increased lymphocyte and decreased neutrophil percentages at the high dose. Untargeted serum metabolomics revealed distinct exposure-related metabolic profiles, including 18 putatively annotated signature metabolites and 26 differentially abundant metabolites. Bilirubin and presqualene diphosphate were exclusively detected in exposed animals, whereas metabolites associated with lipid oxidation and mitochondrial fatty acid β-oxidation were elevated after exposure. RT-qPCR further supported altered expression of genes involved in these pathways. Overall, PS-MPs disrupted hematological homeostasis and metabolic regulation, likely through hepatic lipid peroxidation and systemic inflammation, and serum bilirubin and presqualene diphosphate may serve as candidate biomarkers of exposure.

## 1. Introduction

Micro/nanoplastics (MNPs) are recognized as persistent emerging contaminants that threaten ecosystems. Studies indicate that humans may ingest 0.1–5 g of MNPs (<1 mm) weekly through diet and other pathways (e.g., inhalation and dermal contact), equating to an estimated 74,000–121,000 particles annually [1,2,3]. Direct evidence of MNP accumulation in human tissues is mounting, with the highest burdens observed in the respiratory system, followed by the circulatory system, and with females showing higher accumulation than males [4]. Exposure to MNPs can damage the nervous, respiratory, and immune systems, inducing tissue oxidative stress, inflammation, and metabolic alterations [5,6,7]. MNPs have also been detected in the mammalian reproductive system, where they are associated with reproductive toxicity [8]. Despite growing awareness of their toxicity, extracting and quantifying MNPs from biological matrices remain technically difficult [9,10]. Therefore, there is an urgent need to identify simple and reliable candidate biomarkers for assessing MNPs exposure.

Pregnancy is a critical window of susceptibility, during which environmental exposures can profoundly influence outcomes. Subtle maternal disturbances may be amplified, affecting placental function, immune tolerance, and fetal developmental programming [11,12,13]. The detection of MNPs in the placenta, amniotic fluid, and breast milk has raised significant concerns about their potential risks to maternal and fetal health [14,15,16,17]. Blood is the central conduit linking the mother and fetus. Disruptions in maternal blood homeostasis can directly impair placental perfusion and fetal oxygen supply; maternal anemia or hypoxia is closely associated with adverse outcomes like fetal growth restriction and preterm birth [18,19,20]. Blood homeostatic imbalance often involves elevated inflammatory mediators and oxidative stress, which may compromise placental function and increase pregnancy risks [21,22]. Such dysregulation is also implicated in complications like preeclampsia and gestational diabetes, further affecting fetal development [23,24]. Thus, maintaining blood homeostasis is vital. Notably, MNP accumulation in the human circulatory system is estimated at 0.8–7.36 mg [4], yet whether this exposure disrupts blood homeostasis and fetal development remains unknown.

Polystyrene (PS), a widely used polymer, is common in everyday items such as foam packaging and disposable utensils [25,26]. Polystyrene microplastics (PS-MPs) originate from primary production or product degradation [27]. Due to their prevalence, varied particle sizes, and ability to adsorb co-contaminants, PS-MPs are a major focus of toxicological research. Importantly, particle size is a key determinant of microplastic bioavailability because it influences gastrointestinal uptake, systemic distribution, and the ability to cross biological barriers. Experimental studies in mammals have shown that smaller PS particles generally exhibit greater biodistribution after oral exposure. For example, compared with 5 µm PS-MPs, 0.5 µm PS-MPs show broader organ distribution, whereas 5 µm particles are still detectable in serum and tissues such as the liver, brain, and kidney [28]. Likewise, direct comparison of 100 nm, 3 µm, and 10 µm PS particles demonstrated that the nanosized particles entered the blood and multiple tissues more readily than the larger microplastic fractions [29]. In pregnancy-related models, both 5 µm PS-MPs and 50 nm PS-NPs induced placental dysfunction, but the hemodynamic disturbances were more pronounced for the smaller particles, further supporting the importance of size in maternal–fetal risk assessment [30]. PS-MPs can elevate reactive oxygen species (ROS) and cause oxidative DNA damage in multiple organs [31,32,33]. Regarding reproductive toxicity, PS-MPs disrupt the maternal–fetal immune balance [34]. Animal studies indicate that maternal ingestion of high doses of MNPs during pregnancy or lactation can cause premature offspring death and developmental defects [8]. PS-MPs can cross the placenta, reach fetal brain tissue, and induce neurobehavioral abnormalities [35,36]. These findings indicate that early-life exposure to PS-MPs causes maternal and offspring toxicity, in which the circulatory system is likely to play a key role. Based on the above evidence, we selected 1 µm PS-MPs for the main exposure experiment because this size lies at the lower end of the microplastic range and is more likely than larger MPs to undergo intestinal uptake and systemic distribution while still representing true microplastic exposure. In addition, 5 µm fluorescent PS microspheres were used only in the tracing experiment to facilitate visualization of maternal and fetal tissue distribution.

Current research on microplastics health effects focuses largely on acute or organ-specific toxicity (e.g., gut, liver, and reproduction), with limited evidence on systemic blood homeostasis and metabolic regulation [37,38,39]. Therefore, we established a mouse model of gestational and lactational PS-MPs exposure to investigate tissue accumulation (particularly in blood) and toxic effects. We further employed serum metabolomics in pregnant mice to identify exposure-related metabolic alterations, aiming to provide a basis for screening clinically applicable blood candidate biomarkers for PS-MP exposure.

## 2. Materials and Methods

### 2.1. Chemicals

Two types of PS-MPs were purchased from Beisile Chromatography Technology Development Center (Tianjin, China). Green fluorescent PS microspheres (5 µm in diameter; cat. no. 7-3-0500) with excitation/emission wavelengths of 488/518 nm were used for qualitative assessment of tissue accumulation. Non-fluorescent PS-MPs (1 µm in diameter; cat. no. 6-1-0100) were used for in vivo exposure experiments. Both materials were supplied as aqueous suspensions (1% and 2.5% *w*/*v*, respectively). Before administration, the PS-MP stock suspension was first sonicated for 10 min to re-disperse the particles, then diluted with deionized water to the target concentration and sonicated again for 10 min. All working suspensions were freshly prepared before administration and gently vortexed before each intragastric gavage administration to minimize particle sedimentation. Since no quantitative characterization of the particles in the diluted suspension was performed in this study, this treatment procedure was intended to minimize, rather than completely eliminate, particle aggregation.

### 2.2. Scanning Electron Microscope (SEM) Imaging

The morphology and size of PS-MPs were characterized using a scanning electron microscope (SEM, Thermo Scientific Apreo 2C, Brno, Czech Republic). Briefly, suspensions were drop-cast onto silicon wafers and air-dried overnight in a fume hood. Dried samples were then sputter-coated with a thin gold layer for 1 min using a Polaron SC7640 sputter coater (Quorum Technologies Ltd., Lewes, UK) prior to observation under field-emission SEM (FE-SEM).

### 2.3. Animals and Experimental Design

Six- to seven-week-old C57BL/6J mice were purchased from Xiamen Fudexin Biotechnology Co., Ltd. (Xiamen, China). All animal procedures were approved by the Institutional Animal Care and Use Committee of Fujian Agriculture and Forestry University (Ethics Approval No. PZCASFAFU24136) and were conducted in accordance with relevant guidelines. Mice were housed under standard laboratory conditions (22 ± 2 °C, 55 ± 15% relative humidity, a 12 h light/dark cycle, and ventilation ≥ 15 times/h) with ad libitum access to food and water.

After a 2-week acclimatization period, females were co-housed with males overnight for mating at a 2:1 ratio. The presence of a vaginal plug the following morning was designated as gestational day 0 (GD0). Plug-positive females were then randomly assigned to one of the following four groups (6–7 per group): a control group (control, Con) receiving ultrapure water, and three exposure groups receiving PS-MPs (1 µm) via oral gavage at low (LD, 0.4 mg/kg bw/day), medium (MD, 4 mg/kg bw/day), or high (HD, 40 mg/kg bw/day) doses. Gavage administration continued daily from GD0 until postnatal day 21 (PND21, weaning). Intragastric gavage was performed in the morning at a volume of 10 mL/kg, adjusted based on the current body weight of each maternal mouse before each administration. A ball-tipped stainless steel gavage needle (8#45 mm, Globalebio, Beijing, China) was used. All gavage procedures were carried out by the same operator who had received training from the animal facility. Prior to administration, the needle length was verified according to the animal’s body size; no forced insertion was applied if resistance was encountered. During and after the gavage, reflux, coughing, dyspnea, oronasal bleeding, and other procedure-related discomforts were monitored. No gavage-related adverse events were observed during the study. Maternal body weight, water intake, and food consumption were recorded every 3 days. For the main cohort, the day of birth was recorded as PND0, and litter size was counted. Pregnancy and delivery outcomes are summarized in Table S1. Blood samples from dams in the Con and HD groups were collected for serum metabolomics analysis (LC-MS). On PND21, after a 12 h fast, dams and offspring were anesthetized with isoflurane and euthanized for terminal sample collection (blood and liver).

To evaluate the maternal–fetal transfer and tissue distribution of PS-MPs, an additional independent cohort of pregnant mice was established for fluorescence tracing analysis. In this tracing cohort, mice were orally administered 5 µm green fluorescently labeled PS-MPs by daily intragastric gavage from GD0 to GD18. The mice were euthanized on GD18, and samples were collected. Peripheral blood was collected from both dams and fetuses to prepare blood smears. In addition, liver, brain, and intestinal tissues were harvested, embedded in OCT compound, and subjected to frozen sectioning for fluorescence localization observation.

**Figure 1:**
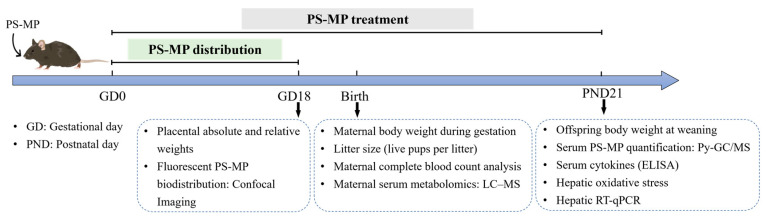
Experimental design for assessing the effects of gestational and lactational exposure to PS-MPs in mice. Pregnant C57BL/6J mice (GD0) were randomly assigned to the following four groups: Con, LD (0.4 mg/kg bw/day), MD (4 mg/kg bw/day), and HD (40 mg/kg bw/day), and were orally gavaged with 1 μm PS-MPs or ultrapure water. The main exposure experiment was conducted from GD0 to PND21 to evaluate growth and developmental outcomes, hematological alterations, hepatic oxidative stress, inflammatory responses, and serum metabolomic profiles in dams and offspring. The day of birth was designated as PND0, and terminal samples were collected on PND21. In addition, an independent tracing experiment was performed in which pregnant mice were orally administered 5 μm fluorescently labeled PS-MPs from GD0 to GD18. Maternal and fetal blood and tissue samples were collected on GD18 to assess the tissue distribution and maternal–fetal transfer of PS-MPs.

Overall, this study employed a continuous maternal exposure model from GD0 to PND21 to evaluate the combined effects of gestational and lactational exposure on dams and offspring, while a separate GD0–GD18 tracing cohort was used to assess maternal-fetal transfer and tissue distribution. The experimental timeline is summarized in Figure 1.

### 2.4. Preparation of Frozen Tissue Sections

Liver, brain, and intestinal tissues collected on GD18 were fixed in 4% paraformaldehyde for 24 h. Tissues were then trimmed, sequentially cryoprotected in 15% and 30% sucrose solutions at 4 °C until they sank, embedded in optimal cutting temperature (OCT) compound, and rapidly frozen. Serial sections (8–10 µm thick) were cut using a cryostat, mounted onto clean glass slides, labeled, and stored at −20 °C until analysis.

### 2.5. Confocal Imaging of Fluorescently Labeled PS-MPs

Frozen tissue sections (liver, brain, and intestine) and peripheral blood smears from dams and fetuses collected on GD18 were imaged using a confocal laser scanning microscope (Leica STELLARIS 5, Mannheim, Germany). Image acquisition parameters (laser power, gain, and exposure) were kept consistent for all samples of the same tissue type. Unexposed control tissues were imaged under identical settings to identify and account for autofluorescence. For each section, 6–8 random fields of view were captured to ensure a representative assessment of fluorescent signal distribution.

### 2.6. Identification and Quantification of Microplastics in Blood

Whole blood samples from the high-dose (HD) exposed dams and their offspring (*n* = 3 per group) were processed for quantification of PS-MPs. Briefly, 100 µL of whole blood was mixed with 400 μL of ice-cold extraction solvent (methanol:acetonitrile = 1:1, *v*/*v*; containing internal standards). After vortexing, samples were extracted by low-temperature ultrasonication (5 °C, 40 kHz) for 30 min, incubated at −20 °C for 30 min to precipitate proteins, and centrifuged at 13,000× *g* for 15 min at 4 °C. The supernatant was collected, evaporated to dryness under a gentle stream of nitrogen, and reconstituted in 100 μL of acetonitrile:water (1:1, *v*/*v*). PS-MPs were quantified using pyrolysis-gas chromatography-mass spectrometry (Py-GC/MS). The reconstituted extract was concentrated to dryness at 80 °C, transferred onto Py-GC/MS sample cups, and further heated at 80 °C to ensure complete solvent removal before instrumental analysis. Procedural blanks were processed in parallel. A calibration curve was constructed using polystyrene standards for quantification.

### 2.7. Oxidative Stress Assays

Liver tissues from dams and offspring were homogenized in ice-cold phosphate-buffered saline (PBS, 1:9 *w*/*v*). The homogenates were centrifuged at 4 °C, and the supernatants were collected for biochemical assays. The activities of superoxide dismutase (SOD), catalase (CAT), and the concentration of malondialdehyde (MDA) were measured using commercial assay kits (Beyotime Biotechnology, Shanghai, China) following the manufacturer’s instructions. SOD activity was determined using the WST-8 colorimetric method by measuring absorbance at 450 nm; activity was calculated based on the inhibition rate and normalized to protein content (U/mg protein). CAT activity and MDA content were assayed following the kit protocols, with readout wavelengths and calculations performed as specified by the manufacturer, and results normalized to protein concentration or tissue weight as appropriate. Total protein concentration was determined using a bicinchoninic acid (BCA) protein assay kit for normalization. All assays were performed in triplicate.

### 2.8. RT-qPCR

Total RNA was extracted from the liver tissues using a commercial RNA extraction kit (Aikerui, Changsha, China). cDNA was synthesized from 1 µg of total RNA using the Hifair^®^ III 1st Strand cDNA Synthesis Kit (Yeasen, Shanghai, China). The resulting cDNA was used as the template for RT-qPCR to quantify target gene expression. Primer sequences are listed in Table S2. Quantitative real-time PCR (qPCR) was performed on a LightCycler^®^ system using SYBR Green chemistry (Roche Diagnostics GmbH, Mannheim, Germany). The relative expression of target genes was calculated using the 2^−ΔΔCt^ method, with *Gapdh* serving as the internal reference gene.

### 2.9. Cytokine Measurement by ELISA

Serum levels of tumor necrosis factor-alpha (TNF-α), interleukin-1 beta (IL-1β), interleukin-6 (IL-6), and interleukin-10 (IL-10) were measured using the corresponding mouse enzyme-linked immunosorbent assay (ELISA) kits (KGI Biotech, Nanjing, China) according to the manufacturer’s protocols. A standard curve was generated for each plate. Standards and samples were assayed in triplicate. For offspring, serum from pups within the same litter was pooled, and the litter was treated as the statistical unit.

### 2.10. Complete Blood Count Analysis

On PND0, maternal blood was collected from the retro-orbital plexus into EDTA-coated anticoagulant tubes. Complete blood counts, including a five-part white blood cell differential, were performed immediately using an automated hematology analyzer (BC-5000Vet, Mindray, Shenzhen, China).

### 2.11. Serum Metabolomics Analysis

For metabolomic profiling, serum samples from the control and high-dose dams (*n* = 5 per group) were processed. The high-dose group showed the clearest overall toxicological phenotype in this study. A 100 µL aliquot of serum was mixed with 400 μL of extraction solvent (methanol:acetonitrile = 1:1, *v*/*v*) containing internal standards (e.g., L-2-chlorophenylalanine, 0.02 mg/mL). After vortexing and ultrasonication, samples were incubated at −20 °C and centrifuged at 13,000× *g* and 4 °C. The supernatant was dried under nitrogen and reconstituted in 100 μL of acetonitrile:water (1:1, *v*/*v*). LC–MS analysis was conducted on a Thermo Scientific UHPLC–Q Exactive HF-X system (Thermo Fisher Scientific, Bremen, Germany) equipped with an ACQUITY UPLC HSS T3 column (100 mm × 2.1 mm, 1.8 µm; Waters, Milford, MA, USA). The mobile phases consisted of (A) water:acetonitrile (95:5, *v*/*v*) with 0.1% formic acid and (B) acetonitrile:isopropanol:water (47.5:47.5:5, *v*/*v*/*v*) with 0.1% formic acid. The flow rate was 3 μL/min, and the column temperature was maintained at 40 °C. Mass spectrometry data were acquired in both positive and negative electrospray ionization (ESI) modes. Sheath gas and auxiliary gas were set to 50 and 13 arbs, respectively; heater and capillary temperatures were 425 °C and 325 °C, respectively; spray voltage was ±3500 V; and normalized collision energy (NCE) was set at 20/40/60. Full MS and MS/MS resolutions were 60,000 and 7500, respectively.

Quality control (QC) samples were prepared by pooling equal volumes of each sample extract and were injected at regular intervals (every 5 injections) throughout the run to monitor system stability. Raw data were processed using Progenesis QI software v3.0 (Waters, USA) for peak picking, alignment, and normalization. Metabolites were annotated by matching MS/MS spectra against the HMDB, Metlin, and in-house databases. Orthogonal partial least squares-discriminant analysis (OPLS-DA) was employed to discriminate between groups. Metabolites with a variable importance in projection (VIP) score > 1.0 and a *p*-value < 0.05 (Student’s *t*-test) were considered differentially abundant. Pathway analysis was performed based on the Kyoto Encyclopedia of Genes and Genomes (KEGGs) database.

### 2.12. Data Analysis

Data are presented as mean ± standard error of the mean (SEM). Statistical analyses were performed using GraphPad Prism software (version 8.3; San Diego, CA, USA). Differences between two groups were assessed using an unpaired two-tailed Student’s *t*-test. Comparisons among multiple groups were analyzed by one-way analysis of variance (ANOVA) followed by Tukey’s post hoc test for multiple comparisons. For data involving repeated measures over time (e.g., body weight), two-way ANOVA with Šidák’s multiple comparisons test was used. Statistical significance was set at *p* < 0.05.

## 3. Results

### 3.1. PS-MPs Are Absorbed into Systemic Circulation and Undergo Transplacental Transfer

**Figure 2:**
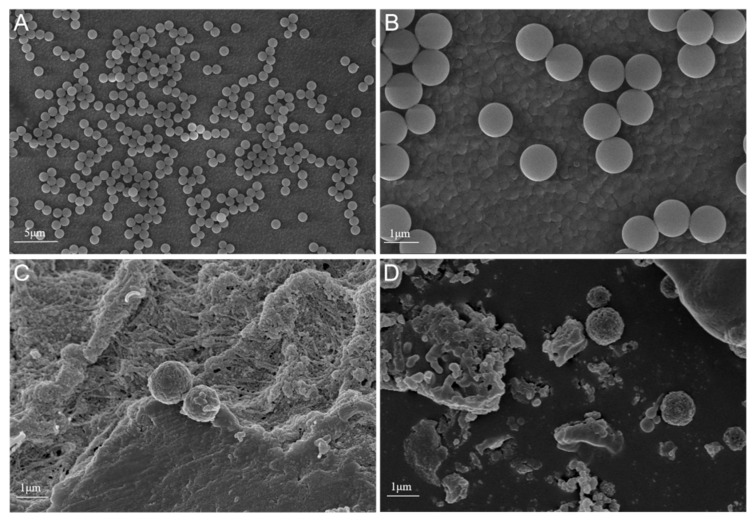
Morphological characterization of PS-MPs and evidence of their intestinal absorption and systemic distribution. (**A**,**B**) Representative SEM images of pristine PS-MPs (1 µm) at different magnifications, showing their spherical morphology, smooth surface, and uniform size distribution. Scale bars: (**A**) 5 µm, (**B**) 1 µm. (**C**) SEM image of intestinal mucosal tissue from a PS-MP-exposed dam at GD18, showing PS-MP particles adhering to the tissue surface. Scale bar: 1 µm. (**D**) SEM image of peripheral blood from a PS-MP-exposed dam at GD18, demonstrating the presence of spherical PS-MP particles among blood cells. Scale bar: 1 µm.

We first characterized the PS-MPs used in this study. SEM images showed that the particles were spherical with smooth surfaces and well dispersion, with an average diameter of approximately 1 µm (Figure 2A,B). Following oral exposure, PS-MP particles were observed adhering to the intestinal mucosal surface of pregnant mice (Figure 2C) and in peripheral blood (Figure 2D and Table S1), indicating their translocation across the intestinal barrier and into the circulatory system.

A separate experiment using 5 µm fluorescently labeled PS-MPs was conducted to trace their in vivo distribution. Confocal microscopy revealed distinct green fluorescence signals in the intestinal, hepatic, and brain tissue sections of exposed dams (Figure S2A–C). Critically, similar fluorescence signals were detected in the corresponding tissues of fetuses (intestine, liver, and brain), supporting maternal-to-fetal transfer and organ-associated distribution of PS-MPs (Figure S2D–F).

**Figure 3:**
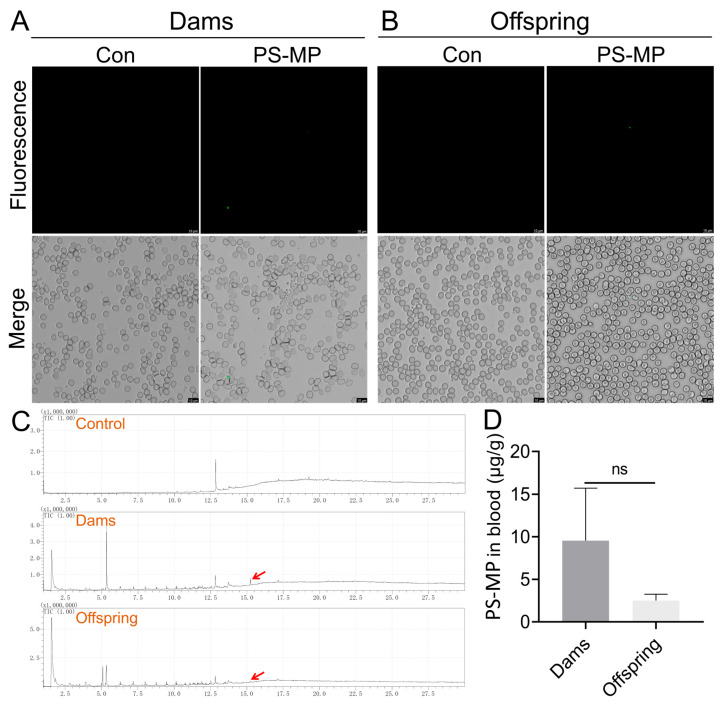
Quantitative and qualitative detection of PS-MPs in maternal and offspring blood. (**A**,**B**) Representative confocal microscopy images showing green fluorescent PS-MPs microspheres (5 µm) in blood smears from (**A**) a GD18 dam and (**B**) a GD18 fetus following maternal exposure. Scale bars: 20 µm. (**C**) Representative total ion chromatograms (TICs) from Py-GC/MS analysis of blood extracts. The characteristic styrene trimer peak (retention time ~10.5 min) is present in samples from exposed dams and offspring but absent in procedural blanks. The red arrows indicate the characteristic fragment peak of the styrene trimer detected in blood samples from exposed dams and offspring. (**D**) Quantification of PS-MP concentrations in whole blood from high-dose exposed dams and their offspring at GD18, as determined by Py-GC/MS. Data are presented as mean ± SEM (*n* = 3 dams; for offspring, *n* = 3 litters with one fetus analyzed per litter). Statistical comparisons between dams and offspring were performed using an unpaired two-tailed Student’s *t*-test (ns, not significant).

PS-MPs were also detected in maternal and offspring blood (Figure 3A,B). To quantitatively confirm the presence and intergenerational transfer of PS-MPs in blood, we employed Py-GC/MS. Total ion chromatograms showed characteristic fragment peaks of styrene trimers in blood samples from exposed dams and offspring, which were absent in procedural blanks (Figure 3C). Quantitative analysis indicated that the mean PS-MPs concentration was 9.56 ± 6.17 µg/g in maternal blood and 2.51 ± 0.14 µg/g in offspring blood; the concentration in dams was 3.8-fold higher than that in offspring (Figure 3D). Together, these results indicate that orally administered PS-MPs are absorbed, distributed to maternal tissues and peripheral blood, and are transferred across the placenta to offspring, albeit with a lower burden in fetal circulation.

### 3.2. PS-MP Exposure Induces Hepatic Oxidative Stress and Affects Pregnancy Outcomes

**Figure 4:**
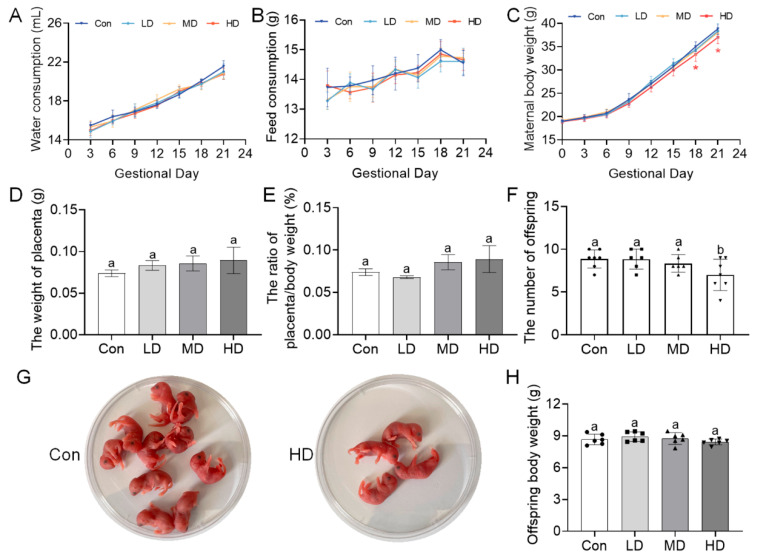
Maternal PS-MP exposure from gestation through lactation alters maternal physiology and offspring developmental outcomes. Pregnant mice were orally administered PS-MPs at doses of 0 (control), 0.4 (LD), 4 (MD), or 40 (HD) mg/kg bw/day from GD0 to PND21. (**A**) Daily water consumption and (**B**) daily feed consumption of dams during gestation (GD0 to GD21). (**C**) Maternal body weight change throughout gestation. Data in (**A**–**C**) are presented as mean ± SEM (*n* = 6–7 dams per group) and were analyzed by two-way ANOVA with Šidák’s multiple comparisons test (* *p* < 0.05 for the HD group vs. control from GD18 onward). (**D**) Absolute placental weight and (**E**) relative placental weight (placental weight/maternal body weight at GD18) of fetuses collected at GD18. (**F**) Number of live pups per litter at birth (PND0). (**G**) Representative photograph of newborn pups from control and HD groups at PND0. (**H**) Body weight of offspring at weaning (PND21). For panels (**D**–**H**), the litter was treated as the statistical unit. Data are presented as mean ± SEM (*n* = 3–7 litters per group as indicated). Statistical significance was determined by one-way ANOVA followed by Tukey’s post hoc test. Different lowercase letters above bars denote significant differences (*p* < 0.05) between groups. In panels (**F**,**H**), the overlaid symbols indicate individual samples, with circles for Con, squares for LD, upward triangles for MD, and downward triangles for HD.

To assess systemic toxicity of PS-MPs, we monitored the effects of exposure from gestation through lactation on growth and development. Throughout gestation, maternal water intake (Figure 4A) and food consumption (Figure 4B) did not differ significantly between exposed and control groups (*p* > 0.05), suggesting that PS-MP exposure did not alter basic ingestive behavior. However, maternal body weight gain during late gestation was significantly affected; from GD18 onward, the HD group exhibited significantly lower body weight compared to the Con group (*p* < 0.05) (Figure 4C). At GD18, placental weight and relative placental weight (placenta/body weight) showed an increasing trend with dose but did not reach statistical significance (Figure 4D,E). Litter size decreased with increasing PS-MP dose, and the HD group had significantly fewer live pups per litter than the Con group (*p* < 0.05) (Figure 4F,G). At weaning (PND21), offspring body weight did not differ significantly from controls (*p* > 0.05) (Figure 4H). These results indicate that PS-MP exposure impaired maternal weight gain and reduced litter size.

**Figure 5:**
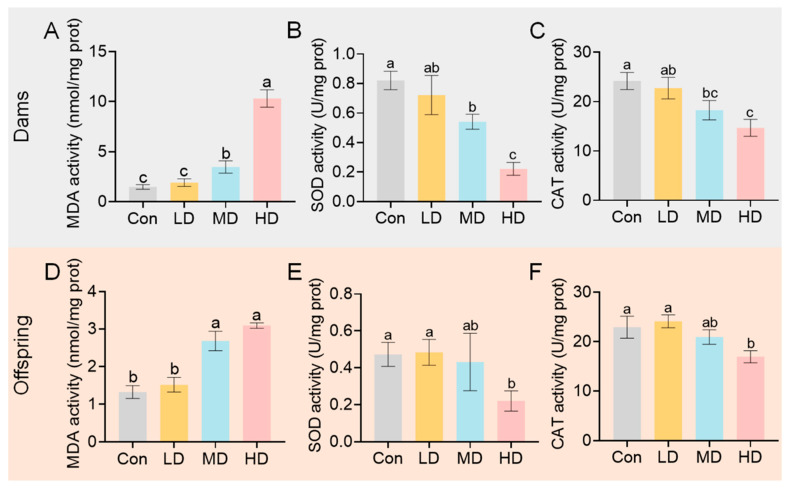
Hepatic oxidative stress indices in dams and offspring following PS-MP exposure. (**A**–**C**) Levels of MDA and activities of SOD and CAT in the livers of dams at PND21. (**D**–**F**) Levels of MDA and activities of SOD and CAT in the livers of offspring at PND21. Pregnant mice were orally administered PS-MPs at doses of 0 (control), 0.4 (LD), 4 (MD), or 40 (HD) mg/kg bw/day from GD0 to PND21. Liver tissues were collected at the time of euthanasia for biochemical assays. For offspring, tissues from pups of the same litter were pooled, with the litter treated as the statistical unit. Data are presented as mean ± SEM (*n* = 3 dams or litters per group). Statistical significance was determined by one-way ANOVA analysis by Tukey’s post hoc test. Different lowercase letters above bars denote significant differences (*p* < 0.05) between groups.

Given the central role of the liver in xenobiotic metabolism and redox regulation, we evaluated hepatic oxidative status. The liver index (liver weight/body weight) showed no significant differences among groups in either dams or offspring (Figure S3). However, biochemical assays revealed the following significant changes: MDA levels were significantly increased in the MD and HD groups in both dams and offspring (Figure 5A,D). Conversely, the activities of the antioxidant enzymes SOD and CAT were significantly decreased in these groups (Figure 5B,C,E,F). These findings indicate that PS-MP exposure disrupts hepatic redox homeostasis, promoting lipid peroxidation while impairing antioxidant defense.

**Figure 6:**
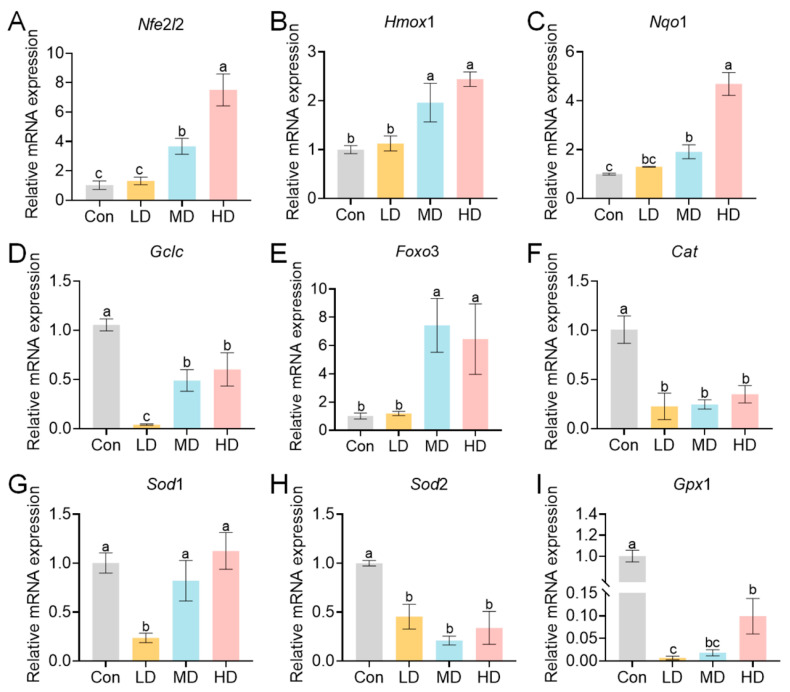
Hepatic expression of oxidative stress-related genes in dams exposed to PS-MPs. Pregnant mice were orally administered PS-MPs at doses of 0 (control), 0.4 (LD), 4 (MD), or 40 (HD) mg/kg bw/day from GD0 to PND21. (**A**–**I**) Relative mRNA expression levels (normalized to *Gapdh*) of oxidative stress pathway genes: (**A**) *Nfe2*l*2* (*Nrf*2), (**B**) *Hmox*1, (**C**) *Nqo*1, (**D**) *Gclc*, (**E**) *Foxo*3, (**F**) *Cat*, (**G**) *Sod*1, (**H**) *Sod*2, and (**I**) *Gpx*1. Liver tissues were collected at PND21 for RNA extraction and qPCR analysis. Data are presented as mean ± SEM (*n* = 3 dams per group). Statistical significance was determined by one-way ANOVA analysis by Tukey’s post hoc test. Different lowercase letters above bars denote significant differences (*p* < 0.05) between groups.

To elucidate the molecular mechanisms underlying this oxidative stress, we analyzed the expression of key genes. In maternal livers, exposure induced a dose-dependent upregulation of genes in the Nrf2/ARE antioxidant pathway (Figure 6). The expression of the core transcription factor *Nfe*2*l*2 was significantly upregulated in the MD and HD groups compared with the Con group, reaching its peak in the HD group (*p* < 0.01) (Figure 6A). Its downstream targets *Hmox*1 and *Nqo*1 were also significantly upregulated in the MD and HD groups (*p* < 0.01) (Figure 6B,C). The expression of *Gclc* was significantly decreased in the LD group, then increased with dose but remained below control levels (*p* < 0.01) (Figure 6D). The oxidative stress regulator *Foxo*3 was significantly upregulated in MD and HD (*p* < 0.01) (Figure 6E). In contrast, the expression of genes encoding antioxidant enzymes, including *Cat*, *Sod*2, and *Gpx*1, was significantly downregulated in all exposed groups relative to Con (*p* < 0.01) (Figure 6F,H,I). *Sod*1 expression was significantly downregulated only in the LD group (*p* < 0.01) (Figure 6G).

**Figure 7:**
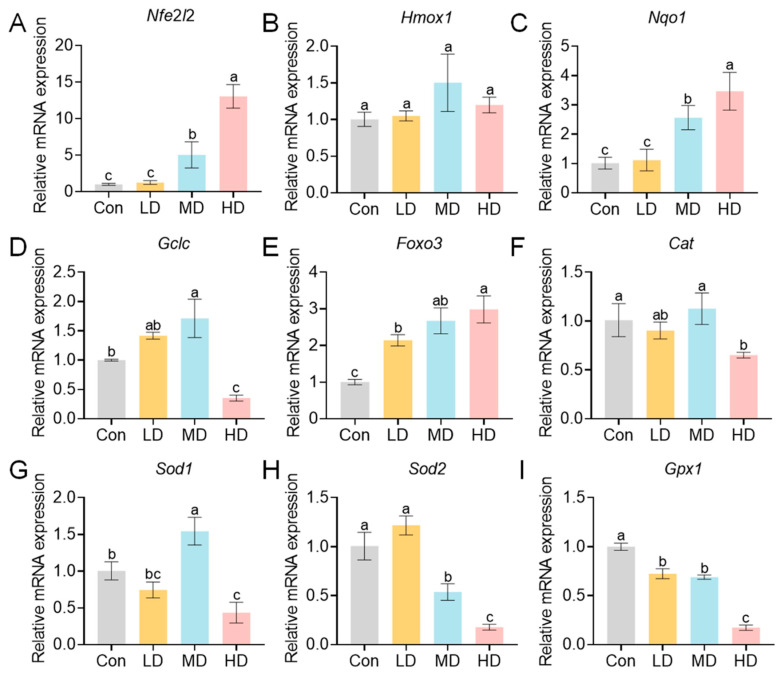
Hepatic expression of oxidative stress-related genes in offspring exposed to PS-MPs. Offspring mice were exposed to PS-MPs via maternal oral gavage at doses of 0 (control), 0.4 (LD), 4 (MD), or 40 (HD) mg/kg bw/day from GD0 to PND21. (**A**–**I**) Relative mRNA expression levels (normalized to *Gapdh*) of oxidative stress pathway genes: (**A**) *Nfe*2*l*2 (*Nrf*2), (**B**) *Hmox*1, (**C**) *Nqo*1, (**D**) *Gclc*, (**E**) *Foxo*3, (**F**) *Cat*, (**G**) *Sod*1, (**H**) *Sod*2, and (**I**) *Gpx*1. Liver tissues were collected at PND21 for RNA extraction and qPCR analysis. Offspring from the same litter were pooled for analysis, with the litter treated as the statistical unit. Data are presented as mean ± SEM (*n* = 3 litters per group). Statistical significance was determined by one-way ANOVA analysis by Tukey’s post hoc test. Different lowercase letters above bars denote significant differences (*p* < 0.05) between groups.

A similar pattern was observed in offspring livers, where PS-MP exposure activated oxidative stress-related pathways (Figure 7). *Nfe*2*l*2 expression increased with dose, showing significant upregulation in MD and HD compared to Con (*p* < 0.05) (Figure 7A). The expression levels of *Nqo*1 and *Foxo*3 expressions also increased with dose (Figure 7C,E), whereas *Hmox*1 showed no significant change (*p* > 0.05) (Figure 7B). *Gclc* expression was significantly higher in the MD than in the Con but was significantly downregulated in the HD (*p* < 0.05) (Figure 7D). *Cat* and *Sod*1 were significantly downregulated in the HD group (*p* < 0.05) (Figure 7F,G). *Sod*2 and *Gpx*1 were significantly downregulated in both MD and HD groups (*p* < 0.05) (Figure 7H,I). These results demonstrate that medium- to high-dose PS-MP exposure induces hepatic oxidative stress in offspring and suppresses the expression of key antioxidant enzymes.

### 3.3. PS-MP Exposure Disrupts Maternal Hematological Homeostasis and Remodels the Serum Metabolome

**Figure 8:**
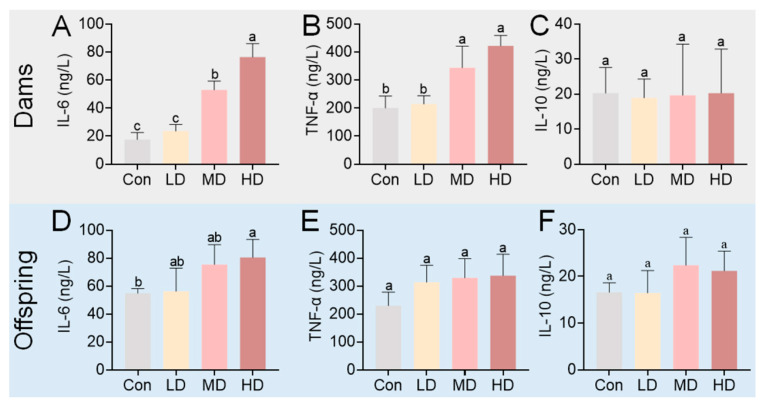
Serum levels of inflammatory cytokines in dams and offspring following PS-MP exposure. (**A**–**C**) Levels of IL-6, TNF-α, and IL-10 in dams at PND0. (**D**–**F**) Levels of IL-6, TNF-α, and IL-10 in offspring at PND21. Pregnant mice were orally administered PS-MPs at doses of 0 (control), 0.4 (LD), 4 (MD), or 40 (HD) mg/kg bw/day from GD0 to PND21. Blood samples were collected from dams at PND0 and from offspring at PND21 for cytokine quantification by ELISA. Offspring serum samples were pooled by litter, with the litter treated as the statistical unit. Data are presented as mean ± SEM (*n* = 4 dams or litters per group). Statistical significance was determined by one-way ANOVA followed by Tukey’s post hoc test. Different lowercase letters above bars denote significant differences (*p* < 0.05) between groups within the same panel.

Having established that PS-MPs transfer to offspring via circulation and induce oxidative stress, we further investigated their effects on hematological parameters and systemic metabolism. Serum levels of inflammatory cytokines were measured by ELISA (Figure 8). Compared to the Con group, maternal serum concentrations of IL-6 and TNF-α were significantly elevated in the MD and HD groups (*p* < 0.01), while the increase in the LD group was not statistically significant (*p* > 0.05) (Figure 8A,B). Maternal IL-10 levels did not differ among groups (*p* > 0.05) (Figure 8C). In offspring, IL-6 was significantly elevated in the HD group, while TNF-α and IL-10 levels showed no significant changes (*p* > 0.05) (Figure 8D–F). These findings indicate a dose-dependent pro-inflammatory response in dams, primarily characterized by elevated IL-6 and TNF-α, whereas offspring exhibited a significant increase only in IL-6 levels at the highest dose.

**Figure 9:**
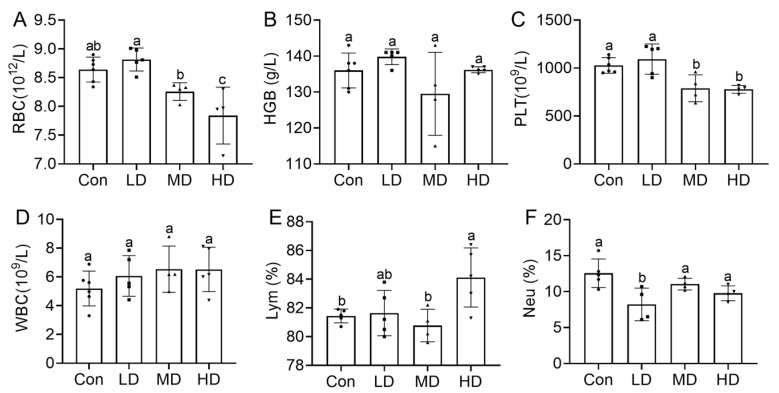
Hematological alterations in pregnant dams following gestational exposure to PS-MPs. (**A**) Red blood cell (RBC) count. (**B**) Hemoglobin (HGB) concentration. (**C**) Platelet (PLT) count. (**D**) White blood cell (WBC) count. (**E**) Percentage of lymphocytes (LYM%). (**F**) Percentage of neutrophils (NEU%). Blood samples were collected on PND0 for complete blood count analysis. Data are presented as mean ± SEM (*n* = 4–6 dams per group). Statistical significance was determined by one-way ANOVA followed by Tukey’s post hoc test. Different lowercase letters above bars denote significant differences (*p* < 0.05) between groups. The overlaid symbols indicate individual samples, with circles for Con, squares for LD, upward triangles for MD, and downward triangles for HD.

Complete blood count analysis of dams on PND0 revealed significant alterations (Table S3). RBC counts exhibited a decreasing trend, with a significant reduction in the HD group compared to Con (*p* < 0.01) (Figure 9A). HGB concentration did not differ significantly (Figure 9B). PLT counts were significantly reduced in both MD and HD groups (*p* < 0.05) (Figure 9C). WBC counts showed an increasing trend but did not reach statistical significance (*p* > 0.05) (Figure 9D). The leukocyte differential was altered: the lymphocyte percentage (Lym%) was significantly increased in the HD group (*p* < 0.05) (Figure 9E), while the neutrophil percentage (Neu%) was significantly lower in the LD group (*p* < 0.05) (Figure 9F). Additionally, mean platelet volume (MPV) in the LD group and eosinophil count (Eos#) and hematocrit (HCT) in the MD group also differed significantly from Con (*p* < 0.05) (Table S3). Overall, PS-MP exposure was associated with reduced RBC and PLT counts and an altered leukocyte profile in dams.

**Figure 10:**
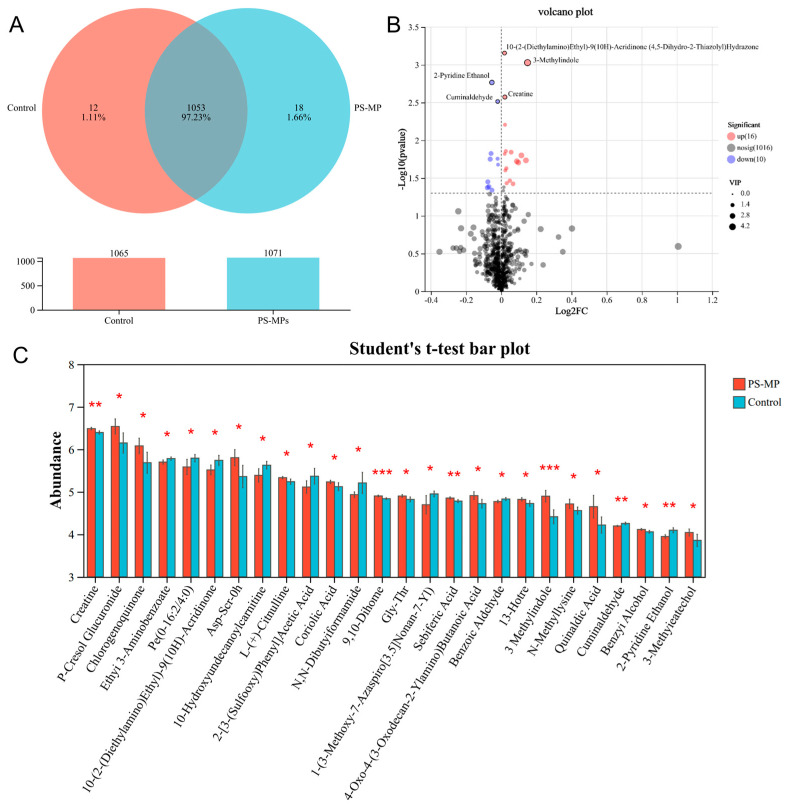
Serum metabolomic profiling reveals significant perturbations in pregnant dams exposed to PS-MPs. (**A**) Venn diagram illustrating the number of metabolites detected in serum from control (Con) and high-dose (HD, 40 mg/kg bw/day) PS-MP-exposed dams. Unique and overlapping metabolites are shown. (**B**) Volcano plot of differentially abundant metabolites between Con and HD groups. Each point represents a metabolite. Red points indicate significantly upregulated metabolites (log_2_(fold change) > 0 and −log_10_(*p*-value) > 1.3), blue points indicate significantly downregulated metabolites (log_2_(fold change) < 0 and −log_10_(*p*-value) > 1.3), and gray points represent non-significant metabolites. Dashed lines mark the thresholds for statistical significance (*p* = 0.05) and a fold-change of 2. (**C**) Heatmap of the relative abundance (Z-score normalized) of the 26 significantly differential metabolites between the Con and PS-MPs groups. Data are presented as mean ± SEM (*n* = 5 dams per group). Statistical significance for individual metabolites was assessed by an unpaired Student’s *t*-test (* *p* < 0.05, ** *p* < 0.01, *** *p* < 0.001).

To gain a comprehensive, system-level understanding of the impact of PS-MP exposure on the maternal blood milieu, we performed untargeted serum metabolomics. Orthogonal partial least squares-discriminant analysis (OPLS-DA) showed clear separation between the Con and HD groups in both positive and negative ion modes (Figure S4A). A sample correlation heatmap also indicated low within-group variation and distinct between-group differences (Figure S4B). Venn diagram analysis putatively annotated 1053 shared metabolites common to both the Con and HD groups, along with group-specific metabolites (12 unique to Con, 18 unique to HD). Notably, features putatively annotated as bilirubin and presqualene diphosphate were observed in the HD group under our annotation criteria (Figure 10A and Table S4). Using criteria of variable importance in projection (VIP) > 1 and *p* < 0.05, we obtained 26 differentially abundant metabolite features with putative annotations between Con and HD, including 16 upregulated and 10 downregulated (Figure 10B). In the HD group, several lipid oxidation-related metabolites (13-HOTrE, 9,10-DiHOME, sebiferic acid, and coriolic acid) were increased. Metabolites associated with mitochondrial fatty acid β-oxidation (10-hydroxyundecanoylcarnitine) and creatine were also elevated. Changes in amino acid metabolism and protein methylation were evident, with L-citrulline, Gly-Thr, Asp-Ser-OH, and N-methyllysine predominantly increased. The gut co-metabolite 3-methylindole and the phase II detoxification metabolite p-cresol glucuronide were also upregulated. Conversely, a decrease in PE(O-16:2/4:0) suggested potential alterations in membrane phospholipid composition (Figure 10C and Figure S4C).

**Figure 11:**
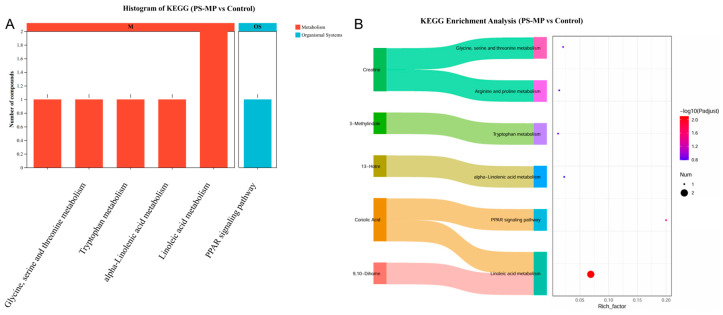
Pathway enrichment analysis of serum metabolites altered by PS-MP exposure. (**A**) Histogram displaying the top enriched KEGG pathways based on the differential metabolites putatively annotated between control and PS-MP-exposed dams. The *X*-axis represents the pathways, and the *Y*-axis lists the number of compounds. The colors of the bars represent the following classification of the pathways: red for metabolism and blue for organismal systems. (**B**) KEGG pathway enrichment scatter plot. Each bubble represents a metabolic pathway. The *X*-axis indicates the enrichment factor (Rich Factor), the *Y*-axis lists the pathway names, the bubble size corresponds to the number of differential metabolites mapped to that pathway, and the bubble color reflects the −log_10_(*p*-value) of the enrichment significance. Key annotated pathways are labeled.

KEGG enrichment analysis revealed that the differential metabolites were primarily enriched in pathways related to amino acid metabolism, α-linolenic acid metabolism, linoleic acid metabolism, and the PPAR signaling pathway (Figure 11A). For instance, creatine was mapped to glycine, serine and threonine metabolism, and arginine and proline metabolism; 3-methylindole to tryptophan metabolism; 13-HOTrE to α-linolenic acid metabolism; coriolic acid to PPAR signaling; and 9,10-DiHOME to linoleic acid metabolism (Figure 11B). These pathways represent the major metabolic networks perturbed by PS-MP exposure. Importantly, these pathway-level changes were closely aligned with the oxidative-stress phenotype observed in the liver. The increased levels of 13-HOTrE, 9,10-DiHOME, and coriolic acid indicate enhanced oxidation and remodeling of polyunsaturated fatty-acid-derived lipid mediators after PS-MP exposure. Together with the elevated hepatic MDA levels and reduced antioxidant enzyme activities, these findings support a mechanistic link between oxidative stress and the disruption of lipid-related pathways, particularly α-linolenic acid metabolism, linoleic acid metabolism, and PPAR-associated lipid signaling.

**Figure 12:**
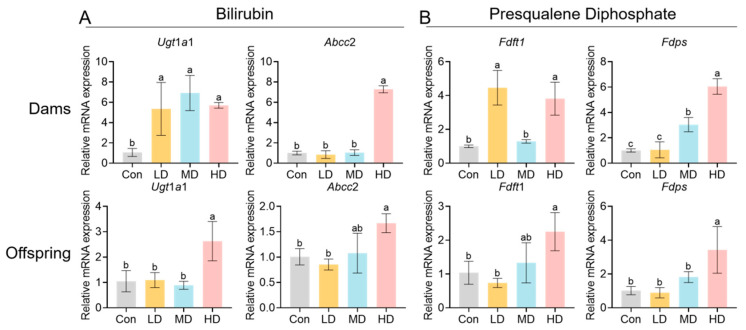
Expression levels of genes associated with selected PS-MP exposure-specific metabolites in the livers of dams and offspring. After exposure-specific metabolites were putatively annotated from serum metabolomics, qPCR was performed to quantify core genes in the relevant pathways in the livers of dams and offspring. Results are presented as relative mRNA expression levels. (**A**) Bilirubin: expression changes in *Ugt*1*a*1 and *Abcc*2 in the livers of dams and offspring. (**B**) PSDP (presqualene diphosphate): expression changes in *Fdft*1 and *Fdps* in the livers of dams and offspring. Data are shown as mean ± SEM (*n* = 3 dams or 3 litters per group). Statistical significance was determined by one-way ANOVA-followed analysis by Tukey’s post hoc test. Different lowercase letters above bars denote significant differences (*p* < 0.05) between groups.

Transcriptional validation was further performed for selected PS-MP exposure-specific metabolites and differentially abundant metabolites. Specifically, qPCR was used to verify the expression of key genes in pathways associated with the exposure-specific metabolites bilirubin and presqualene diphosphate, including *Ugt*1*a*1 and *Abcc*2, as well as *Fdft*1 and *Fdps*. The results showed that *Ugt*1*a*1 exhibited an overall increasing trend across all exposure groups in dams, with a significant increase observed in the HD group of offspring; *Abcc*2 was significantly upregulated in the HD group in both dams and offspring (Figure 12A). In the livers of dams, *Fdft*1 was elevated in the LD and HD groups, whereas *Fdps* displayed a dose-dependent upward trend and reached the highest level in the HD group; in offspring livers, *Fdft*1 and *Fdps* were also significantly upregulated in the HD group (Figure 12B).

**Figure 13:**
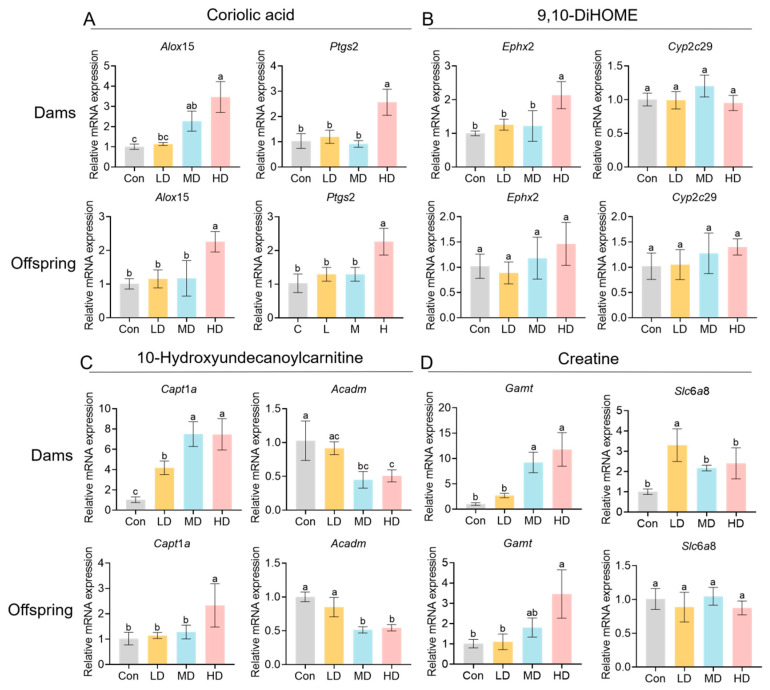
Expression levels of genes associated with differentially abundant metabolites in the livers of dams and offspring. Following the screening and putative annotation of differentially abundant metabolite features from serum metabolomics, qPCR was performed to quantify core genes in the corresponding pathways in the livers of dams and offspring. Results are presented as relative mRNA expression levels. (**A**) Coriolic acid: expression changes in *Alox*15 and *Ptgs*2 in the livers of dams and offspring. (**B**) 9,10-DiHOME: expression changes in *Ephx*2 and *Cyp*2*c*29 in the livers of dams and offspring. (**C**) 10-hydroxyundecanoylcarnitine: expression changes in *Cpt*1*a* and *Acadm* in the livers of dams and offspring. (**D**) Creatine: expression changes in *Gamt* and *Slc*6*a*8 in the livers of dams and offspring. Data are shown as mean ± SEM (*n* = 3 dams or 3 litters per group). Statistical significance was determined by one-way ANOVA analysis followed by Tukey’s post hoc test. Different lowercase letters above bars denote significant differences (*p* < 0.05) between groups.

Compared with the Con group, the serum levels of the lipid peroxidation-related metabolites coriolic acid and 9,10-DiHOME were elevated in exposed dams. qPCR analysis of genes involved in their associated pathways showed consistent expression patterns of *Alox*15 and *Ptgs*2 in the livers of both dams and offspring, with significant upregulation in the HD group (Figure 13A). By contrast, *Ephx*2 was significantly upregulated only in dams in the HD group, whereas no obvious change was observed in offspring; *Cyp*2*c*29 showed no significant differences between dams and offspring (Figure 13B). In addition, increased serum 10-hydroxyundecanoylcarnitine and creatine in the exposed groups suggested potential alterations in mitochondrial fatty acid β-oxidation and energy metabolism. qPCR results indicated that Acadm was significantly downregulated in the MD and HD groups in the livers of both dams and offspring, whereas *Cpt*1*a* exhibited an overall upward trend in both, reaching significance only in the offspring HD group (Figure 13C). Among creatine-related genes, hepatic *Gamt* was significantly upregulated in the HD group in both dams and offspring (and was also increased in dams in the MD group), while *Slc*6*a*8 showed dose-related fluctuations only in dams (increased in the LD group but decreased in the MD and HD groups), with no significant differences observed in offspring (Figure 13D).

## 4. Discussion

Microplastics are pervasive environmental contaminants found in aquatic, terrestrial, and atmospheric systems. Their transfer through food webs leads to sustained human exposure and bioaccumulation [40,41,42]. Animal studies confirm that ingested microplastics can cross the intestinal barrier, enter systemic circulation, and accumulate in tissues such as blood, liver, and brain [29,43,44]. During pregnancy, blood serves as the critical conduit between mother and fetus and is a principal route for potential fetal exposure to xenobiotics. Maintaining blood homeostasis is therefore essential for oxygen and nutrient delivery to the developing fetus. The gestational and lactational periods are characterized by dynamic remodeling of fetal immune, metabolic, and hematopoietic systems, rendering them exceptionally sensitive to environmental insults. However, the technical challenges associated with detecting and quantifying microplastics in biological matrices, coupled with the subtle, chronic nature of their toxicity, complicate risk assessment and pose a latent threat to maternal and infant health. In this study, we established a long-term PS-MP exposure model in C57BL/6J mice (from GD0 to PND21) to evaluate transgenerational toxicity and its impact on the blood internal milieu. Our findings demonstrate that PS-MPs accumulated in both dams and offspring, impaired developmental outcomes, elicited hepatic stress, and were accompanied by enhanced systemic inflammation, altered blood cell profiles, and significant shifts in serum metabolites. These results provide a foundational basis for identifying candidate biomarkers indicative of PS-MP exposure.

Recent evidence confirms the presence of diverse microplastics in human lungs, intestines, and blood [41,45,46]. Their detection in pregnancy-associated matrices such as placenta, breast milk, and amniotic fluid has escalated concerns regarding potential risks to maternal–fetal health [14,15,16]. By tracing PS-MPs in vivo, we detected particles in maternal intestine, liver, brain, and blood, with corresponding fluorescence signals in fetal tissues, supporting absorption, systemic distribution, and maternal-to-offspring transfer (Figure 3, Figures S1 and S2). However, because the tracing sub-study employed 5 µm fluorescently labeled PS-MPs, whereas the main toxicity experiment used 1 µm non-fluorescent PS-MPs, these localization data should be interpreted as qualitative supportive evidence rather than an exact representation of the biodistribution pattern of the 1 µm particles. Moreover, this study did not include vascular perfusion, endothelial co-localization, or in-section chemical confirmation of polymer identity. Accordingly, the fluorescence signals observed in the brain and liver sections cannot be regarded as definitive evidence that PS-MPs had crossed the blood–brain barrier or penetrated the tissue parenchyma. Future studies incorporating vascular labeling, three-dimensional imaging, and Raman/FTIR-based polymer confirmation would be valuable for resolving the precise microanatomical localization of PS-MPs in these organs. Despite these limitations, the observed maternal-to-offspring transfer is consistent with previous reports of gestational microplastic translocation across the placental barrier and related offspring phenotypes [35,47,48,49].

At the same time, previous studies indicate that smaller polystyrene micro/nanoplastic particles generally exhibit stronger barrier-crossing ability, broader tissue biodistribution, and greater toxic potential than larger particles, although the relationship is not strictly linear across all endpoints [28,50]. Therefore, the 5 µm tracing results do not substitute for, but also do not argue against, the biological plausibility that the smaller 1 µm particles used in the main experiment could enter the circulation and undergo maternal-to-offspring transfer. Quantitative Py-GC/MS analysis further corroborated the presence of PS in blood, with maternal concentrations exceeding those in offspring by approximately 3.8-fold (Figure 3C,D), likely reflecting a partial filtering function of the placenta [46]. In addition, SEM observation of peripheral blood revealed scattered particles with a relatively regular spherical morphology and a diameter of approximately 1 µm (Figure 2D), providing morphological support that the 1 µm particles used in the main experiment crossed the intestinal barrier and entered systemic circulation.

Importantly, offspring outcomes observed in this study cannot be attributed solely to direct particle exposure. Although PS-MP-related signals were detected in fetal tissues and offspring blood, maternal systemic inflammation, oxidative stress, and metabolic disturbances may also indirectly affect offspring outcomes through changes in the intrauterine environment and placental function [21,22,23,24,30,34,48]. Moreover, as breast milk was not analyzed, the contribution of lactational transfer remains unclear. The offspring alterations reported here should therefore be interpreted as arising from combined direct and indirect effects across gestation and lactation, rather than from a single causal pathway.

Having established PS-MP translocation, we assessed its effects on pregnancy outcomes and offspring development. While maternal ingestive behavior remained unchanged, high-dose exposure suppressed body weight gain during late gestation (Figure 4A–C). However, because maternal body weight was not measured immediately after parturition and the high-dose group also showed a reduced litter size, the lower body weight observed in late gestation cannot be attributed solely to altered maternal energy metabolism and may partly reflect a reduced fetal burden. Nevertheless, this observation remains broadly consistent with previous reports suggesting that PS-MPs may disturb maternal metabolic homeostasis and increase the risk of adverse pregnancy outcomes [48,51,52]. Furthermore, although placental mass was unaffected, litter size was significantly reduced in the high-dose group (Figure 4D–G). Despite the absence of a detectable change in placental mass, previous studies suggest that microplastics may still induce placental structural and functional abnormalities, potentially compromising early gestational processes like decidualization and implantation, thereby reducing embryonic viability [53]. Offspring body weight and liver index at weaning (PND21) were comparable to controls, consistent with some studies in which offspring phenotypes were not markedly altered under specific exposure conditions [51,54]. Because maternal exposure continued until PND21, offspring phenotypes in the present study should be interpreted as reflecting combined prenatal and postnatal maternal exposure, including potential lactational and early postnatal indirect/direct exposure, rather than effects attributable exclusively to gestational exposure. Future studies using separated exposure windows will be needed to disentangle the relative contributions of prenatal and postnatal exposure.

Based on the FDA body-surface-area conversion approach [55], the mouse doses of 0.4, 4, and 40 mg/kg bw/day correspond approximately to human-equivalent doses of 0.032, 0.324, and 3.24 mg/kg/day, or about 13.6, 136.2, and 1362.2 mg/week for a 60 kg adult. Although this comparison should be interpreted cautiously, given the strong influence of particle size, shape, polymer type, and surface properties on microplastic toxicokinetics, the present doses appear to exceed currently estimated average background exposure levels for the general population. At the same time, the middle- and high-dose HEDs still fall within the broad mass-based intake range of 0.1–5 g/week proposed in a widely cited estimate, whereas the low dose is slightly below that range [3]. Therefore, the present dosing design is best regarded as an elevated-to-high experimental exposure framework for hazard identification and mechanistic evaluation during sensitive periods such as gestation and lactation, rather than a direct simulation of routine background human exposure or one exact real-world internal exposure scenario.

The maintenance of blood homeostasis is a systemic process involving multiple organs, with the liver playing a central role in detoxification, coagulation factor synthesis, and inflammation regulation [56,57]. Our study revealed that PS-MP exposure significantly increased hepatic MDA levels and decreased SOD and CAT activities in both dams and offspring, indicating heightened lipid peroxidation and compromised antioxidant defenses (Figure 5). This was accompanied by activation of the Nrf2/ARE pathway at the transcriptional level, yet a paradoxical downregulation of key antioxidant enzyme genes (*Cat*, *Sod*2, and *Gpx*1) (Figure 6 and Figure 7). This suggests that the compensatory antioxidant response may be insufficient or dysregulated under sustained exposure, leading to a net loss of redox balance. The lower magnitude of lipid peroxidation in offspring relative to dams may be attributable to their lower tissue burden of PS-MPs. These results reinforce that oxidative stress is a principal mechanism of microplastic toxicity [5,6,7], with hepatic redox imbalance serving as a likely central instigator of downstream systemic effects [31,32,33].

Enhanced hepatic oxidative stress often coincides with pro-inflammatory signaling. Accordingly, we observed a dose-dependent increase in serum pro-inflammatory cytokines (IL-6, TNF-α) in exposed dams and elevated IL-6 in high-dose offspring (Figure 8). IL-6 and TNF-α are pivotal mediators of systemic inflammation and metabolic dysregulation. Their sustained elevation, as reported in chronic PS-NP exposure models [58,59], supports a paradigm of continuous exposure-induced sustained low-grade inflammation. This inflammatory milieu likely interacts with the observed hematological alterations. Specifically, PS-MP exposure was associated with decreased RBC and platelet counts in dams (Figure 9A–C). RBCs and platelets are essential for oxygen transport and hemostasis, respectively, and also participate in inflammatory modulation [60,61,62]. Their coordinated reduction could compromise maternal oxygen delivery and coagulation homeostasis, potentially increasing vulnerability to stressors. While total WBC count was unchanged, the altered leukocyte differential (increased lymphocyte and decreased neutrophil percentages) points to a specific immunomodulatory effect [63,64], possibly linked to the observed inflammatory state (Figure 9D–F). Although we did not directly assess PS-MP burden in hematopoietic organs in the present study, the observed reductions in RBC and platelet counts, together with leukocyte redistribution, suggest that potential involvement of the bone marrow or spleen should be considered. Previous studies have shown that PS-MPs can alter gene expression in mouse bone marrow cells and inhibit hematopoietic colony formation, and that multi-scale PS micro-/nanoplastics can penetrate bone marrow and reduce colony-forming, self-renewal, and differentiation capacity [63,65]. In addition, smaller PS particles have been reported to induce more severe splenic injury in mice [28], and long-term microplastic exposure has also been shown to impair hematopoietic stem cell self-renewal [66]. Therefore, besides systemic oxidative stress and inflammation, potential effects on hematopoietic organs may also contribute to the hematological alterations observed in dams. Future studies should directly determine PS-MP burden, histopathological changes, and hematopoietic consequences in the bone marrow and spleen. Collectively, these hematological changes substantiate the disruption of blood homeostasis and provide a phenotypic link to the underlying inflammation and oxidative processes.

Serum metabolomics offered a system-level view of the PS-MP-induced metabolic perturbation. We putatively annotated 18 metabolites unique to the exposed group, including bilirubin and presqualene diphosphate. Bilirubin is a potent endogenous antioxidant; its elevation may represent a compensatory cytoprotective mechanism, and higher levels have been associated with favorable prognosis in conditions like ischemic stroke [67,68]. Presqualene diphosphate is a direct metabolic intermediate produced in the sterol biosynthesis pathway during the reaction catalyzed by squalene synthase (FDFT1). It also functions as a signaling lipid; during immune cell activation, it can be rapidly remodeled into presqualene monophosphate and is implicated in regulating superoxide anion generation and inflammation-related responses [69,70]. Among the differentially abundant metabolites, those related to lipid peroxidation (13-HOTrE, 9,10-DiHOME, sebiferic acid, and coriolic acid) were notably upregulated (Figure 10), providing a direct molecular correlate to the observed hepatic oxidative damage. KEGG pathway analysis further enriched these findings to lipid metabolism pathways (α-linolenic acid and linoleic acid metabolism) and the PPAR signaling pathway (Figure 11), indicating a broad rewiring of lipid and energy metabolism. Concurrent changes in amino acid metabolism (e.g., L-citrulline, Gly-Thr) suggest a broader disruption of nitrogen and protein metabolism. This metabolomic signature aligns with prior studies showing that microplastic exposure systematically reshapes host metabolism [71,72].

This interpretation is biologically plausible because oxidative stress and lipid metabolic remodeling are tightly interconnected. Elevated MDA reflects enhanced lipid peroxidation, whereas the accumulation of oxidized lipid-related metabolites such as 13-HOTrE, 9,10-DiHOME, and coriolic acid suggests that PS-MP exposure promotes oxidative remodeling of polyunsaturated fatty acid metabolism [73,74,75,76]. Therefore, the metabolomic changes observed in α-linolenic acid metabolism, linoleic acid metabolism, and PPAR signaling may represent downstream metabolic manifestations of the hepatic oxidative-stress response.

Based on the differentially abundant and exposure-specific metabolites putatively annotated from serum metabolomics, we further validated key genes in the relevant pathways at the hepatic transcriptional level. Overall, the results were consistent with the direction of metabolic remodeling suggested by the metabolomic profiles. These findings not only enhance the biological interpretability of the candidate exposure biomarkers but also provide liver-derived molecular evidence supporting alterations in the circulating internal milieu. Taken together, the distinct metabolic profile, particularly the presence of bilirubin and presqualene diphosphate, highlights their potential utility as candidate biomarkers for monitoring PS-MP exposure. However, because untargeted metabolomics is primarily a hypothesis-generating discovery approach and the present comparison was based on a modest sample size (*n* = 5 dams per group), these metabolites should currently be regarded as preliminary candidate exposure-related features rather than validated biomarkers. Further confirmation of these key metabolites in larger independent cohorts, as well as validation using authentic standards and targeted quantitative MS/MS analyses, will be required.

## 5. Conclusions

**Figure 14:**
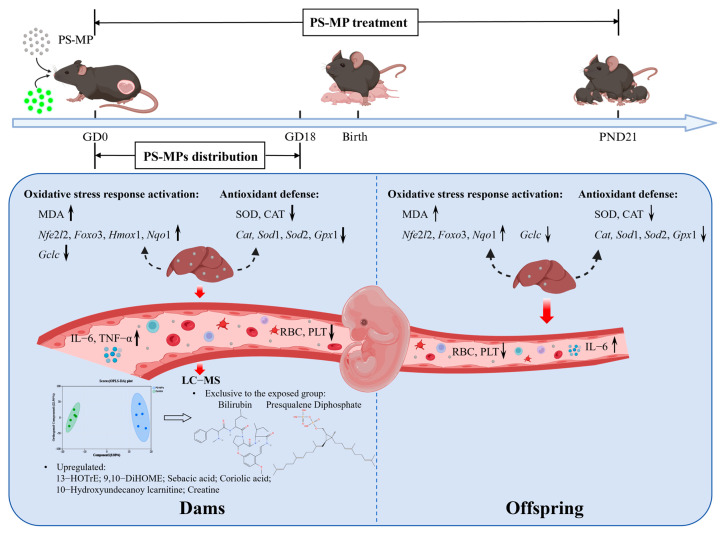
A conceptual model summarizing the transgenerational effects of PS-MP exposure during pregnancy and lactation in mice, including disruption of blood cell homeostasis and metabolic profiles and the potential underlying mechanisms. The figure was drawn by ©2026 BioRender (https://www.biorender.com/; accessed on 20 January 2026).

This study provides a systematic evaluation of the effects of PS-MPs on blood homeostasis in dams and their offspring following maternal exposure from gestation through lactation. Our results indicate that PS-MPs can enter maternal circulation and support the possibility of maternal-to-offspring transfer under the present experimental conditions. This exposure triggered hepatic oxidative stress and systemic inflammatory activation in both generations. Maternal hematological parameters were significantly altered, revealing a disruption in blood cell homeostasis. Serum metabolomic profiling further uncovered a profound remodeling of the maternal metabolic landscape, with the detection of exposure-unique metabolite features putatively annotated as bilirubin and presqualene diphosphate. Differential metabolites were prominently enriched in pathways related to lipid oxidation, including 13-HOTrE, 9,10-DiHOME, sebiferic acid, and coriolic acid. These metabolites represent promising candidate biomarkers for detecting and assessing PS-MP exposure (Figure 14). Our findings advance the understanding of microplastic-induced hematological and metabolic toxicity and establish a foundation for developing blood-based candidate biomarkers to monitor environmental exposure during vulnerable life stages such as pregnancy.

## Data Availability

All the data are contained within the article.

## Supplementary Materials

The following supporting information can be downloaded at https://www.mdpi.com/article/10.3390/toxics14050354/s1, Figure S1: SEM identification of PS-MP in peripheral blood. Representative SEM images of peripheral blood from PS-MP exposed dams at GD18, showing the presence of spherical PS-MP particles (1 µm, indicated by arrows) among blood cellular components. Scale bar: 2 µm; Figure S2: Tissue distribution of fluorescently labeled PS-MP in dams and offspring. Confocal microscopy images showing the accumulation of green fluorescent PS-MP microspheres (5 µm, arrows) in various tissues harvested at GD18 following maternal oral exposure (40 mg/kg bw/day). (A–C) Tissues from exposed dams: (A) intestine, (B) liver, (C) brain. (D–F) Corresponding fetal tissues from the same exposure group: (D) intestine, (E) liver, (F) brain. Scale bars: 25 µm. Tissues from unexposed control animals showed no specific fluorescence under identical imaging settings (not shown); Figure S3: Liver coefficients in dams and offspring at PND21. (A) Liver coefficient (liver weight / body weight × 100%) of dams. (B) Liver coefficient of offspring at weaning. Pregnant mice were orally administered PS-MP at doses of 0 (Control), 0.4 (LD), 4 (MD), or 40 (HD) mg/kg bw/day from GD0 to PND21. For offspring, tissues from pups of the same litter were pooled, with the litter treated as the statistical unit. Data are presented as mean ± SEM (*n* = 6–7 dams or litters per group). Statistical significance was determined by One-way ANOVA followed by Tukey’s post hoc test. Different lowercase letters above bars denote significant differences (*p* < 0.05) between groups; Figure S4: Multivariate and univariate of serum metabolomic profiles between control and PS-MP exposed dams. (A) The overlap and uniqueness of serum metabolites detected in the control (Con) and high-dose PS-MP exposed (HD, 40 mg/kg bw/day) groups based on OPLS-DA. (B) Sample correlation heatmap depicting the pairwise Pearson correlation coefficients between individual serum samples within and between the Con and HD groups. Red indicates high positive correlation; blue indicates low or negative correlation. (C) Hierarchical clustering heatmap displaying the relative abundance (Z-score normalized) of all identified metabolites across samples from the Con and HD groups (*n* = 5 per group). Rows represent metabolites, columns represent individual samples. Color scale indicates relative abundance levels; Table S1: Pregnancy and delivery outcomes following gestational exposure to PS-MPs; Table S2: Primer sequences used for quantitative real-time PCR (qPCR) analysis; Table S3: Hematological profile alterations in pregnant mice exposed to PS-MPs (*n* = 4–6); Table S4: Serum metabolites uniquely detected in the Con group and their mass spectrometric/chromatographic characteristics (*n* = 5); Table S5: Serum metabolites uniquely detected in the PS-MP-exposed group and their mass spectrometric/chromatographic characteristics (*n* = 5).
