# Supplementary material for: Exposure to Polystyrene Microplastics Disrupts Blood Cell Homeostasis and Metabolic Profiles in Pregnant Mice and Offspring: The Role of Oxidative Stress and Inflammation
Source: Toxics. 2026 Apr 23;14(5):354. doi: 10.3390/toxics14050354 (PMC13211503; doi:10.3390/toxics14050354)
Supplement: Supplementary file 1 [file toxics-14-00354-s001.zip › toxics-4222786-supplementary.pdf]

Supplementary Material

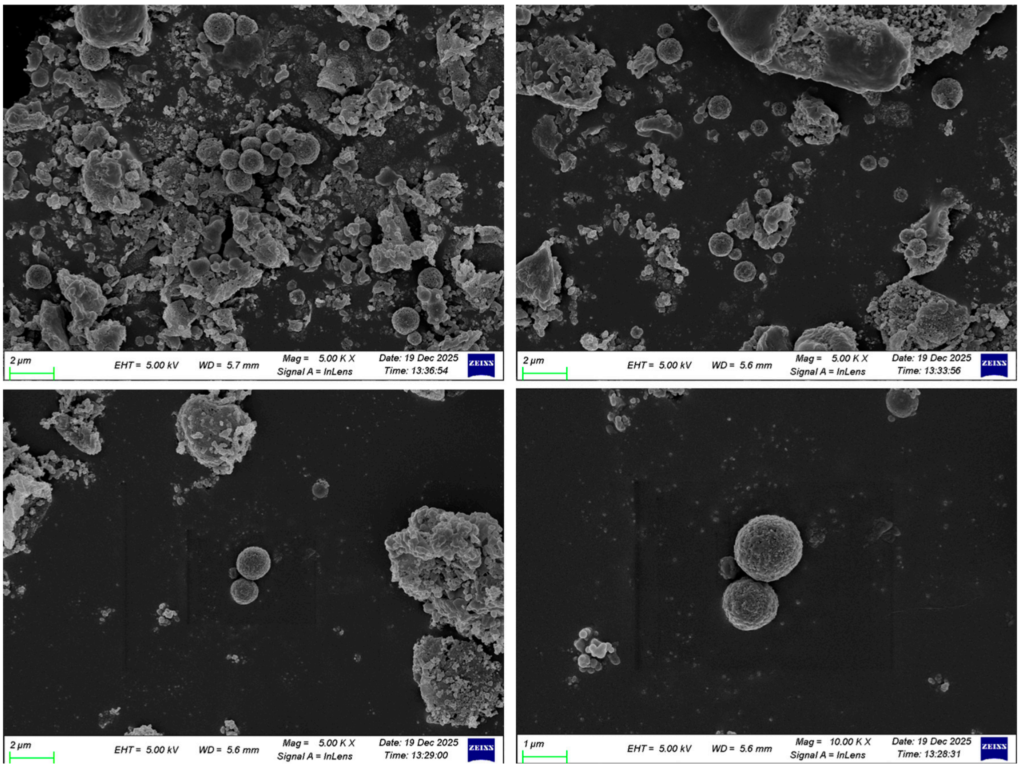

Figure S1. SEM identification of PS-MP in peripheral blood. Representative SEM images of peripheral blood from PS-MP exposed dams at GD18, showing the presence of spherical PS-MP particles (1 μm, indicated by arrows) among blood cellular components. Scale bar: 2 μm.

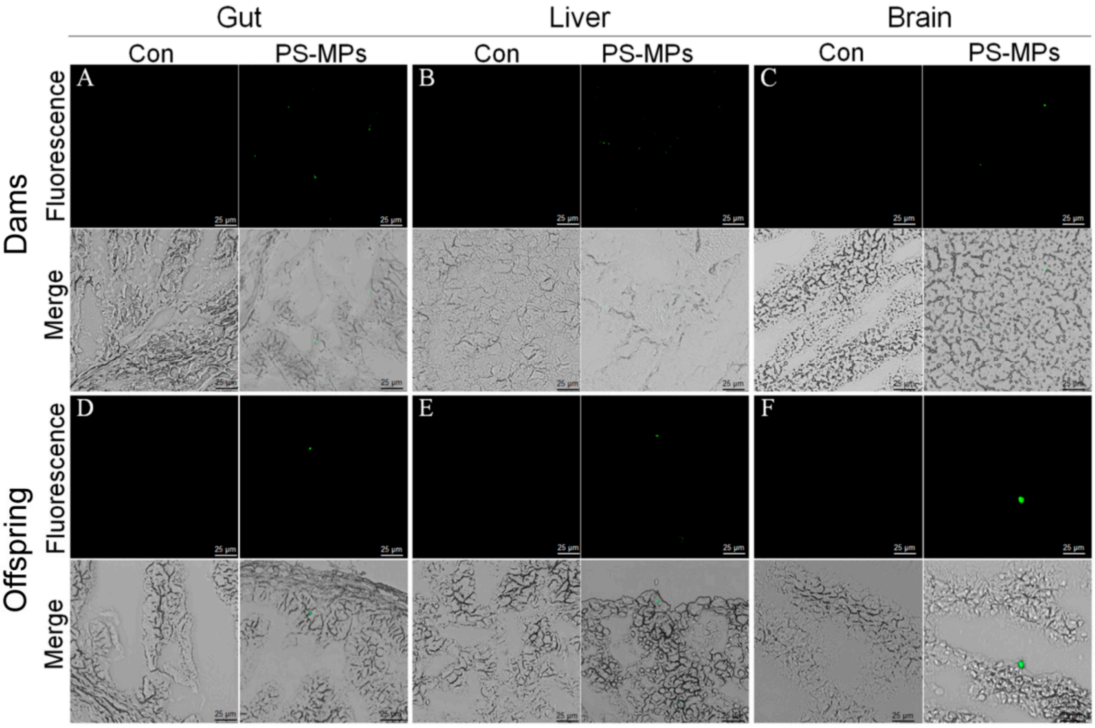

Figure S2. Tissue distribution of fluorescently labeled PS-MP in dams and offspring. Confocal

microscopy images showing the accumulation of green fluorescent PS-MP microspheres (5 μm, arrows) in various tissues harvested at GD18 following maternal oral exposure (40 mg/kg bw/day). (A–C) Tissues from exposed dams: (A) intestine, (B) liver, (C) brain. (D–F) Corresponding fetal tissues from the same exposure group: (D) intestine, (E) liver, (F) brain. Scale bars: 25 μm. Tissues from unexposed control animals showed no specific fluorescence under identical imaging settings (not shown).

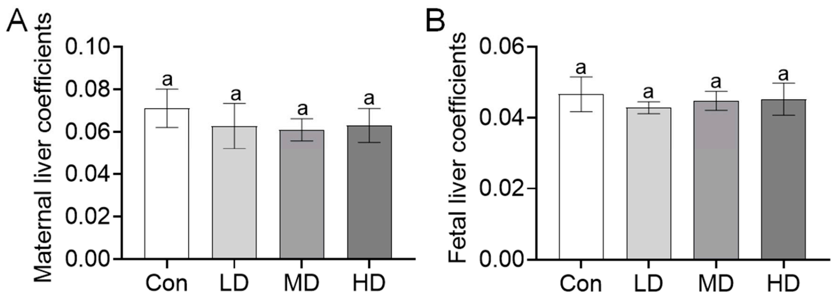

Figure S3. Liver coefficients in dams and offspring at PND21. (A) Liver coefficient (liver weight / body weight × 100%) of dams. (B) Liver coefficient of offspring at weaning. Pregnant mice were orally administered PS-MP at doses of 0 (Control), 0.4 (LD), 4 (MD), or 40 (HD) mg/kg bw/day from GD0 to PND21. For offspring, tissues from pups of the same litter were pooled, with the litter treated as the statistical unit. Data are presented as mean±SEM (n = 6–7 dams or litters per group). Statistical significance was determined by One-way ANOVA followed by Tukey's post hoc test. Different lowercase letters above bars denote significant differences ($p < 0.05$) between groups.

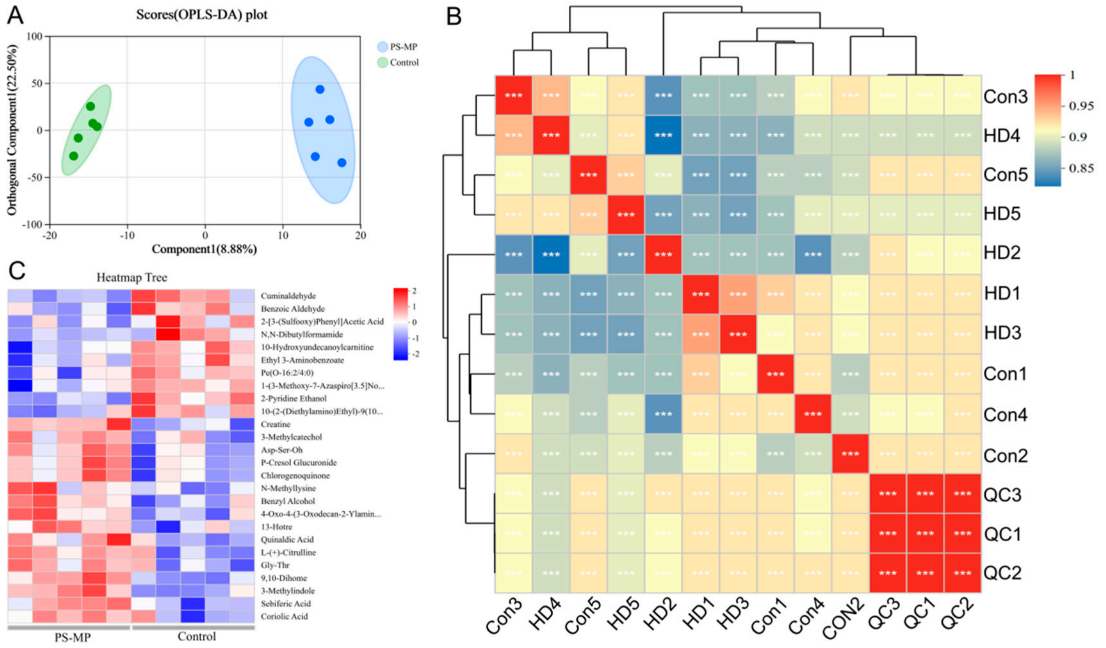

Figure S4. Multivariate and univariate of serum metabolomic profiles between control and PS-MP exposed dams. (A) The overlap and uniqueness of serum metabolites detected in the control (Con) and high-dose PS-MP exposed (HD, 40 mg/kg bw/day) groups based on OPLS-DA. (B) Sample correlation heatmap depicting the pairwise Pearson correlation coefficients between individual serum samples within and between the Con and HD groups. Red indicates high positive correlation; blue indicates low

or negative correlation. (C) Hierarchical clustering heatmap displaying the relative abundance (Z-score normalized) of all identified metabolites across samples from the Con and HD groups (n = 5 per group). Rows represent metabolites, columns represent individual samples. Color scale indicates relative abundance levels.

Table S1. Pregnancy and delivery outcomes following gestational exposure to PS-MP

Group	Vaginal plug checked, n	Successful pregnancies, n	Live-born pups per litter, median (interquartile range, IQR)
Con	8	7	9 (8, 10)
LD	8	6	9 (8, 10)
MD	8	6	8 (8, 9)
HD	8	7	7 (6, 9)

Table S2. Primer sequences used for quantitative real-time PCR (qPCR) analysis

Gene name	Forward Primer	Reverse Primer
Gapdh	CAGGAGAGTGTTTCCTCGTCC	TGAGGTCAATGAAGGGGTCG
Nfe2l2	ACTACAGTCCCAGCAGAGTGATG	GCGTGCTCAGAAACCTCCTTC
Sod2	CTGGAGCCACACATTAACGC	AGTTGTAACATCTCCCTTGGC
Cat	GAGGGATTCCCGATGGTCAC	ATGCCCTGGTCGGTCTTGTA
Gclc	GCACATCTACCACGCAGTCA	GTCTCAAGAACATCGCCTCCA
Hmox1	TGACACCTGAGGTCAAGCAC	CAGCTCCTCAAACAGCTCAATG
Nqo1	TATCACCACTGGGGGTAGCG	CGCAGGATGCCACTCTGAATC
Foxo3	TTGGTGGATCATCAACCCCG	CACTTGGAGAGCTGGGAAGG
Gpx1	ATCAGTTCGGACACCAGAATGG	GGAAGGTAAAGAGCGGGTGAG
Sod1	GGAACCATCCACTTCGAGCA	CCCATGCTGGCCTTCAGTTA
Alox15	CCTGATGACTTGGCTGAG	GCTTGGTCGGTCTTGTAG
Ephx2	GCAGCAAGAAGCATCAAC	GACAGGACTCTATCAGGAAG
Ptgs2	CCATTGACCAGAGCAGAG	CCAGTATTGAGGAGAACAGAT
Cyp2c29	CTGTCTTATTCTCCTCTCACTA	GCTTCCTTCACTGCTTCA
Cpt1a	TCGCACATTACAAGGACAT	CCACATAGAGGCAGAAGAG
Acadm	GACGAAGCCACGAAGTAT	CTCTGTGTTGAATCCATAGC
Gamt	GAGGAACACTGGATTATTGAG	AAGCGGAAGGCATGATTC
Slc6a8	CTTCAACAACAACTGCTACA	CCTGATTCTGCCACCTTG
*Ugt*1*a*1	CCTATGTCAACGCCTCTG	CATCATCACCATCGGAACT
*Abcc*2	TACGGACAACCATCATAGC	GGACCAGGAGAACCATAAG
*Fdft*1	TATAACCTGCTGCGATTCC	TCTTCTTCTCCACACTGATG
Fdps	AGAGTTCCTATCAGACAGAGA	CATTGGCGTGTTCCTTCT

Table S3. Hematological profile alterations in pregnant mice exposed to PS-MP (n = 4-6)

Parameters	Con	LD	MD	HD
WBC (10^9/L)	5.20±1.21	6.07±1.41	6.54±1.61	6.52±1.55
Neu# (10^9/L)	0.60±0.11	0.52±0.21	0.72±0.17	0.47±0.11
Lym# (10^9/L)	4.17±1.10	4.96±1.17	5.29±1.36	5.48±1.25
Mon# (10^9/L)	0.51±0.32	0.57±0.25	0.40±0.12	0.42±0.37
Eos# (10^9/L)	0.06±0.01	0.07±0.04	0.13±0.04*	0.06±0.02
Bas# (10^9/L)	0.01±0.01	0.01±0.00	0.00±0.01	0.01±0.01
Neu%	12.56±1.99	8.23±2.27*	11.05±0.81	9.78±1.03
Lym%	81.44±0.48	81.64±1.58	80.78±1.13	84.12±2.06*
Mon%	8.11±5.09	9.58±6.09	6.20±7.09	6.12±8.09
Eos%	1.21±0.38	1.44±0.77	1.93±0.29	1.06±0.34
Bas%	0.19±0.12	0.16±0.09	0.05±0.06	0.08±0.04
RBC (10^{12}/L)	8.64±0.22	8.82±0.20	8.26±0.15	7.84±0.49*
HGB (g/L)	136.00±4.86	139.80±2.17	129.50±11.56	136.20±0.84
HCT (%)	43.57±0.74	44.50±1.16	40.13±2.68**	42.66±1.41
MCV (fL)	50.56±1.30	50.52±0.99	51.15±0.48	50.96±0.40
MCH (pg)	16.14±0.46	15.86±0.46	16.50±0.53	16.32±0.51
MCHC (g/L)	319.57±8.50	314.20±9.58	322.75±11.50	320.00±9.87
RDW-CV (%)	20.43±2.02	21.46±1.19	21.03±2.63	20.06±1.57
RDW-SD (fL)	42.64±3.81	44.64±2.81	43.75±5.30	41.86±2.79
PLT (10^9/L)	1027.67±81.00	1093.40±157.95	789.25±140.52*	778.50±40.80*
MPV (fL)	6.46±0.26	7.44±0.96*	6.98±0.29	6.65±0.17
PDW	15.41±0.23	15.56±0.25	15.28±0.13	15.32±0.11
PCT (%)	0.69±0.16	0.76±0.12	0.55±0.08	0.57±0.09

Abbreviations: WBC, white blood cell count; Neu#, neutrophil count; Lym#, lymphocyte count; Mon#, monocyte count; Eos#, eosinophil count; Bas#, basophil count; Neu%, neutrophil percentage; Lym%, lymphocyte percentage; Mon%, monocyte percentage; Eos%, eosinophil percentage; Bas%, basophil percentage; RBC, red blood cell count; HGB, hemoglobin; HCT, hematocrit; MCV, mean corpuscular volume; MCH, mean corpuscular hemoglobin; MCHC, mean corpuscular hemoglobin concentration; RDW-CV, red cell distribution width-coefficient of variation; RDW-SD, red cell distribution width-standard deviation; PLT, platelet count; MPV, mean platelet volume; PDW, platelet distribution width; PCT, plateletcrit.

Note: Data are expressed as mean ± SD. *p<0.05 and **P<0.01 indicate statistically significant differences compared with the Con group.

Table S4. Serum metabolites uniquely detected in the Con group and their mass spectro-metric/chromatographic characteristics (*n* = 5)

Metabolite	Metab ID	M/Z	Retention time	Adducts	Formula
Alfaprostolum	metab_2012	407.2783216	6.242	M+H	$C_{24}H_{38}O_5$
Sedanolide	metab_2127	159.1166051	6.3669	M+H-2H$_2$O	$C_{12}H_{18}O_2$
Bufadienolide	metab_2129	319.2415171	6.3669	M+H-2H$_2$O	$C_{24}H_{34}O_2$
Moxidectin	metab_4827	672.4078377	5.0395	M+CH$_3$OH+H	$C_{37}H_{53}NO_8$
norlanostane-trione (epoxy, 29-OH)	metab_2501	507.2479424	6.6329	M+K	$C_{29}H_{40}O_5$
N-(3-Methyl-2-Pyridyl)-3-Phenylsuccinimide	metab_2613	299.1385479	6.9162	M+CH$_3$OH+H	$C_{16}H_{14}N_2O_2$
2-Phenylethyl Octanoate	metab_4222	213.16346	6.3748	M+H-2H$_2$O	$C_{16}H_{24}O_2$
Phe-Asp-Gln	metab_5291	431.1552956	3.0644	M+Na	$C_{18}H_{24}N_4O_7$
Mitemcinal	metab_4780	760.4598236	5.1829	M+Na-H$_2$O	$C_{40}H_{69}NO_{12}$
Benzoylmesaconine	metab_2869	628.2487789	7.6556	M+K	$C_{31}H_{43}NO_{10}$
Enoximone Sulfoxide	metab_11616	299.0261223	6.2293	M+Cl	$C_{12}H_{12}N_2O_3S$
4α-Carboxy-5α-cholesta-8,24-dien-3β-ol	metab_11761	473.3270219	6.1421	M+FA-H	$C_{28}H_{44}O_3$

Abbreviations in table columns: Metab ID, metabolite identifier; M/Z, mass-to-charge ratio; Retention time, chromatographic retention time (min); Adducts, ion adducts observed in mass spectrometry; Formula, molecular formula.

Table S5. Serum metabolites uniquely detected in the PS-MP–exposed group and their mass spectrometric/chromatographic characteristics (*n* = 5)

Metabolite	Metab ID	M/Z	Retention time	Adducts	Formula
Nummularine A	metab_1340	648.3788334	4.2682	M+H	$C_{36}H_{49}N_5O_6$
Tyr-Aib-Aib-Phe-Leu (diallyl-Tyr)	metab_1382	692.405184	4.3842	M+H	$C_{38}H_{53}N_5O_7$
indole–naphthyl ketone (INK)	metab_1430	399.2452585	4.5845	$M+NH_4$	$C_{27}H_{27}NO$
Rphdhd	metab_1523	509.3106473	4.9553	$M+Na-H_2O$	$C_{26}H_{48}O_9$
Flurithromycin	metab_1567	716.4339516	5.1086	$M+H-2H_2O$	$C_{37}H_{66}FNO_{13}$
2,8-Dihydroxyquinoline-Beta-D-Glucuronide	metab_8058	358.0541619	4.3043	M+Na-2H	$C_{15}H_{15}NO_8$
Thr Arg Tyr Asp	metab_3520	554.2538994	7.6712	M+H	$C_{23}H_{35}N_7O_9$
Bilirubin	metab_3782	585.269469	6.9094	M+H	$C_{33}H_{36}N_4O_6$
Silux	metab_4544	554.2748338	6.0473	M+ACN+H	$C_{29}H_{36}O_8$
Digitoxin	metab_4773	782.4729857	5.2092	$M+NH_4$	$C_{41}H_{64}O_{13}$
Sulfolithocholylglycine	metab_2170	514.2823459	6.3826	M+H	$C_{26}H_{43}NO_7S$
3-Indole Carboxylic Acid Glucuronide	metab_12524	336.072378	4.2966	M-H	$C_{15}H_{15}NO_8$
Communesin A	metab_4936	474.2897649	4.5928	$M+NH_4$	$C_{28}H_{32}N_4O_2$
Erythromycin 2'-Acetate	metab_4943	780.4575703	4.5775	$M+Na-H_2O$	$C_{39}H_{69}NO_{14}$
Neamine	metab_4988	355.2190116	4.3842	$M+CH_3OH+H$	$C_{12}H_{26}N_4O_6$
monoterpenol-tetrol-glc	metab_4989	349.1834985	4.3842	$M+H-H_2O$	$C_{16}H_{30}O_9$
Presqualene Diphosphate	metab_5078	604.3525635	4.1441	$M+NH_4$	$C_{30}H_{52}O_7P_2$
Kaltostat	metab_8034	379.0908697	4.2607	$M-H_2O-H$	$C_{14}H_{22}O_{13}$

Abbreviations in table columns: Metab ID, metabolite identifier; M/Z, mass-to-charge ratio; Retention time, chromatographic retention time (min); Adducts, ion adducts observed in mass spectrometry; Formula, molecular formula.
